# Varicella Pneumonia: Case Report and Review of a Potentially Lethal Complication of a Common Disease

**DOI:** 10.1177/2324709618770230

**Published:** 2018-04-18

**Authors:** John T. Denny, Zoe M. Rocke, Valerie A. McRae, Julia E. Denny, Christine Hunter Fratzola, Sajjad Ibrar, Joyce Bonitz, James T. Tse, Shaul Cohen, Scott J. Mellender, Geza K. Kiss

**Affiliations:** 1 Rutgers University, New Brunswick, NJ, USA; 2 St. George’s University, St. George, Grenada; 3 New York University, New York, NY, USA

**Keywords:** varicella, varicella pneumonia, CPAP, radiography of varicella pneumonia, complications of varicella

## Abstract

Varicella zoster virus causes varicella (chickenpox). It can be reactivated endogenously many years later to cause herpes zoster (shingles). Although varicella is usually a benign disease in healthy children, it resulted in over 11 000 hospitalizations and over 100 deaths every year, in all ages, in the United States. Morbidity was considerably worse in older teenagers and adults. Between 5% and 15% of cases of adult chickenpox will produce some form of pulmonary illness. Progression to pneumonia risk factors include pregnancy, age, smoking, chronic obstructive pulmonary disease, and immunosuppression. Typically, pulmonary symptoms occur 1 to 6 days after varicella zoster infection. They often include cough, fever, and dyspnea. Treatment is a 7-day course of intravenous acyclovir for varicella pneumonia. Early intervention may modify the course of this complication. This review illustrates practical features with a case of a 34-year-old female with severe varicella pneumonia. Despite the lack of significant past medical history and absence of immunosuppression, her pneumonia worsened and by using continuous positive airway pressure mask, intubation was avoided. More important, the radiographic progression of severe varicella pneumonia is shown. This highlights how a common disease of varicella can progress in an adult and manifest with significant organ malfunction.

## Introduction

Initial infection with varicella zoster virus (VZV) causes varicella, more commonly known as chickenpox.^1^ VZV, part of the Herpesviridae family, can be reactivated endogenously many years following the initial infection. This secondary infection is known as herpes zoster or shingles. Occurrence of both diseases is worldwide with a relatively low mortality. As a consequence of its high morbidity, VZV puts a significant amount of strain on both the health care system and society as a whole.^2^

## Pathology

The varicella virus can be spread in many ways, but for the most part transmission of VZV is airborne via respiratory droplets. Other ways the virus can be spread is through direct contact with conjunctival fluid, saliva, or fluid from a vesicle of an infected individual. Spread can occur beginning 2 days prior to outbreak of lesions (varicella exanthema). Herpes zoster is less infectious because the only source of infection is the vesicular fluid. Once the vesicular lesion(s) have completely crusted over there is no longer a risk of infection directly from the rash. Initial infection occurs when VZV gains access to regional lymph nodes from mucosa in the upper respiratory tract. VZV infects and replicates within lymph nodes causing a primary viremia within 4 to 6 days of infection. Viral infection spreads to the periphery, infecting mononuclear cells within the blood. Secondary viremia develops within 10 to 14 days of infection and allows for spread to the endothelium of skin capillaries. The virus moves superficially to the epithelium, which triggers inflammation at the site of infection. Vesicles are formed due to the accumulation of tissue fluid following an incubation period of 10 to 21 days.^2^

## Clinical Presentation

Clinically, varicella usually begins with a fever and a rash characterized by pruritus, which affects most of the body and can sometimes spread to mucosal areas. The rash can exist in different stages at the same time on any part of the body, but usually localizes to the face and torso, rather than to the arms and legs. Lesions begin in a macular stage and progress to papules and vesicles, which then crust over within 1 to 2 days. Although the rash is superficial, it often leaves scars or areas with less pigmentation when the crust falls off of each vesicle about 1 to 2 weeks after the rash presents.^3^ Healthy children tend to have self-limiting symptoms on contracting VZV.^1^ Chickenpox is often associated with signs and symptoms of viremia, such as appetite loss, headache, malaise, or fever. In general, cases of varicella are worse when contracted from sick contacts within a household. On the other hand, “breakthrough varicella” is less severe because it occurs in patients who have been immunized against the virus. This means that individuals will have significantly less vesicular lesions and a smaller chance of complications than their nonimmunized counterparts.^3^ Second infections of varicella are unusual.^4^ Lack of immunization, via vaccination or direct infection as a child, as well as old age increases both the morbidity and mortality of varicella in patients.^5^ Prior to vaccination, varicella alone caused upwards of 13 000 hospitalizations and about 150 deaths across all ages per year in the United States of America. Overall, VZV causes mild disease in healthy, younger individuals.^6^ Vaccination is now widely available and many children are vaccinated around 12 months of age to infer lifelong immunity against VZV.^7^

Following an episode of varicella, VZV travels along nerves until it reaches the dorsal root ganglia of the spine where it lies dormant until reactivation years later. Herpes zoster (shingles) is the result of reactivation of VZV.^8^ Both the chickenpox and shingles have similar lesions, but the rashes are distributed differently on the body.^9^ The characteristic rash for shingles is severely painful and remains in the affected dermatome.^8^ Shingles has an increased annual incidence especially after patients surpass the age of 50 years.^10^ Further evidence has stated that about half of 85-year-old individuals report having been affected by herpes zoster at one point in their life. Any type of immunosuppression that affects the cell-mediated portion of a patient’s immune system can also place them at risk of reactivating the varicella virus. This includes but is not limited to infection with human immunodeficiency virus (HIV), cancer, steroid treatment, or diabetes mellitus.^11^ HIV-positive individuals specifically are about 12 to 17 times more likely to have an episode of herpes zoster in their lifetime. In addition, individuals who test positive for shingles in areas with high incidence of HIV have an 85% to 95% chance of having an underlying HIV infection.^11^ Approximately 7 to 25 of 100 000 immunocompromised patients die as a result of developing herpes zoster.^10^

Herpes zoster infections tend to have numerous complications; however, 2 of the most frequent complications are herpes zoster ophthalmicus and postherpetic neuralgia (PHN).^12,13^ Individuals with defective cell-mediated immunity are more at risk for developing PHN or more serious complications like encephalitis.^12^ Herpes zoster is about 5 times more common in patients with hematologic cancers when compared with the general population, and the most common complication in these patients is pain.^12^ Another possible complication, especially in the elderly and immunocompromised, is death, which may be prevented with the administration of both varicella and herpes zoster vaccines. Use of these vaccines in countries that can afford them has shown a fair amount of benefits for at-risk patients.^10^

## Epidemiology

The VZV is distributed globally. However, it is not unusual to have more yearly outbreaks in areas with mild weather in comparison to the tropics. Outbreaks occur more frequently in the later winter months through the spring.^8^ In order to help prevent outbreaks, vaccines like the Oka/Merck have been developed to provide effective immunogenicity for children.^14^ Initially, VZV is highly infectious and can be spread via respiratory droplets, but with reactivation the virus can only be spread via direct contact. Herpes zoster provides a much less efficient mode of transmission, but the latency involved in zoster appears to provide an “evolutionary survival advantage” to VZV.^15^ Respiratory transmission of varicella is supported by evidence of the virus in the oropharynx of infected patients and spread of infection to close contacts prior to the onset of symptoms. In this way it differs from other herpesviruses and can be better compared with measles in terms of infectivity.^16^ Persons living with an individual infected with VZV have a significantly higher risk of contracting the virus in comparison to those exposed to the infection for shorter durations of time. Day care settings, however, have similar transmission rates to those with contacts within the home.^17^ Studies have shown that the most affected age group as far as initial varicella infections is children from 5 to 8 years of age.^18^ In regions with mild temperatures, it is usual for children to become ill with the chickenpox before the age of 10 years. In fact, it is so common in these areas that the incidence of varicella is almost equal to the rate of live births. The United States alone reports over 3 million new varicella cases per year.^8^ Breakthrough varicella has also been reported in about 3% of patients per year who receive the Oka/Merck vaccine against VZV.^14^

Household size is important in the pattern of infection. Nichols et al^19^ demonstrated that in the country of Guinea Bissau, the number of inhabitants per house was a key factor in explaining the early average age of infection. Their data showed a large number of inhabitants per house, on average 3.5 families or 14 individuals, which was the key to understanding the early age of infection despite the low infectivity. The more inhabitants the greater the birth rate, consequently the average size for a newborn’s house is even larger containing 24 individuals, with on average 0.97 births per year. Their model showed that the large number of inhabitants per house in Guinea Bissau is sufficient to explain the early age of infection, comparable to a temperate country, even given the observed within-house rate of infection, which is nearly 5 times lower. Conversely, the later age of infection in other tropical countries can be explained by the smaller household sizes (eg, 4.7-5.5 in Thailand, India, and Singapore).^19^

Incidence of varicella has significantly fallen across all ages following the introduction of the varicella vaccine (Oka/Merck), since 1995. Rates of incidence fell even further after 2006 when a second dose of the vaccine was implemented to improve coverage.^6^ Children are typically vaccinated after 1 year of age. Although statistically insignificant, it has been reported that children vaccinated under 14 months tend to have greater risk of breakthrough illness.^7^

## Complications

In most cases, varicella has a relatively mild course and tends to resolve on its own. However, it is possible for complications to arise regardless of immune status (competent or compromised) prior to infection.^1^ Complications are usually more severe in patients who are pregnant, elderly, or immunocompromised. Infants also have a greater chance of developing more severe complications.^3^ The World Health Organization reported a yearly estimate in 2014 of over 4 million patients requiring hospitalization for severe complications of varicella worldwide.^10^

Similar to other viruses, VZV infection can put a patient at risk of acquiring a secondary viral or bacterial infection.^3^ For this reason, many patients who are at risk for developing severe complications are vaccinated against varicella.^1^ Aside from secondary infections, hematologic, respiratory, neurologic, or renal complications can also occur.^20^ A study of complications in Swiss pediatric patients (vaccine was not recommended at the time of the survey) endorsed that secondary infections with *Staphylococcus aureus* and *Streptococcus pyogenes* were responsible for serious complications in otherwise well children who contracted varicella.^21^ The above-mentioned bacteria usually cause infections of both the skin and subcutaneous tissue. However, in the presence of a previous viral infection progression to a more severe infection is possible, which can include but is not limited to sepsis, osteomyelitis, necrotizing fasciitis, and pneumonia.^3^ Similar infections affected immunocompromised children in the study of Swiss pediatric patients, but this was thought to be due to timely doses of antiviral medication.^21^ As mentioned previously, complications affecting a patient’s neurologic functioning can also occur with varicella. These range from infections affecting a child’s balance to more severe infections of the brain tissue, surrounding meninges, and vasculature. Strokes can also be an associated complication of VZV infection due to inflammation of cerebral blood vessels.^3^ A 2001 study was performed to compare arterial ischemic stroke in children with previous infection with varicella and those without. Evidence showed increased frequency and severity in terms of the region of the brain affected by stroke in children with varicella. There was also an increased risk of recurrent strokes or transient ischemic attacks. These strokes often happen several months after varicella and may fail to be recognized as a complication of the disease.^22^ Other more common and relatively benign complications of varicella include dehydration and decreased PO intake.^3^ The high rate of complications in Swiss pediatric patients challenges the preconceived notion that varicella is a mild disease; thus, immunization against the virus is recommended.^21^

## Pulmonary Complications

Approximately 5% to 15% cases of adult varicella will have a type of respiratory complication.^23^ Pneumonia secondary to varicella infection as well as hospitalization in general seem to occur more frequently in adult populations.^24^ Thus, individuals between the ages of 11 and 15 years who have not had the chickenpox are recommended to receive the varicella vaccination.^1^ Pregnancy, history of pulmonary disease, or smoking, as well as a compromised immune system make patients more susceptible to pulmonary complications of varicella.^25^ The onset of symptoms typically occurs within 1 week of initial symptom presentation of varicella zoster. These include shortness of breath, fever, cough (occasionally hemoptysis), pleuritic chest pain, and signs of hypoxia. Treatment is typically a weeklong course of antivirals administered intravenously.^23^ Varicella pneumonia has a relatively high rate of respiratory failure, but early diagnosis with prompt administration of antiviral medication can improve outcomes.^26^

Radiographically, varicella pneumonia tends to present as bilateral opacifications that appear nodular toward the outer borders and then come together around the base and roots of the lungs. Infiltrate is usually seen across all lung fields.^20,27^

In other case studies of varicella pneumonia, computed tomography scans were performed and found many small, irregular hyperdense abnormalities. Some of the irregularities appeared to combine forming a single larger mass while hazy rings encircled other hyper dense areas.^28^

## Pregnancy

Infection with varicella during pregnancy, especially in the first and second trimesters, can cause a significantly worse varicella infection in infants.^1^ However, pregnancy can also place women at risk for significant complications if infected with varicella. Incidence of varicella-associated pneumonia increases particularly from week 28 of pregnancy to delivery.^26^ The mortality rate of varicella pneumonia increases from 11% in healthy adults to just under 50% in pregnant women. This is due to the increased prevalence of respiratory failure in pregnant women. More often than not, varicella pneumonia causes severe stress due to hypoxia and warrants a cesarean section delivery.^29^ Spontaneous abortion and premature labor can also be seen as complications of varicella infection during pregnancy.^25^ VZV infection in pregnant women either before or after birth can cause serious neonatal infection as well.^1^

As noted above, contracting VZV is very dangerous for both mother and fetus if infection occurs during pregnancy. In terms of dangerous complications to the mother, pneumonia is the most significant concern. However, in infants the more feared complication is congenital varicella, which can occur with infection of the mother between 5 and 24 weeks of pregnancy.^2^ Mortality is relatively high with congenital varicella and symptoms include both brain and eye abnormalities, underdeveloped arms and legs, scarred skin, and some degree of developmental delay.^3^ Unfortunately, varicella in neonates tends to have a worse prognosis than varicella in infants. Neonatal infection occurs anywhere from 5 days prior to birth up until 2 days following the delivery. VZV is transmitted to neonates in 1 of 3 ways: respiratory droplets, through the placenta, or direct contact. Infection is usually fatal due to lack of immune system and no longer having the protection provided by the mother’s immune system in utero.^2^ However, immediate treatment with antivirals and varicella zoster immunoglobulin can greatly increase the survival of newborns with neonatal varicella.^30^

## Immunocompromised Patients

Immunosuppression by way of corticosteroids and chemotherapy has been indicated as a risk factor for severe varicella infections. The virus also has a greater chance of disseminating to other organs in patients who are actively being treated with chemotherapy.^31^ Patients with compromised immune systems generally have worse symptoms and complications. The vesicular rash can become more severe with enlarged vesicles that can become hemorrhagic.^3^ Immune system impairment is one of the major risk factors of developing varicella pneumonia as a complication.^26^ Without a competent immune system, the disease process can quickly develop into adult respiratory distress syndrome followed by overall respiratory failure.^23^ Once the patient is experiencing respiratory failure and must be mechanically ventilated, the mortality rate of varicella pneumonia jumps from about 30% to almost 50% in spite of treatment course.^26^ Overall, complication with varicella pneumonia is the leading cause of death in patients with varicella.^32^

## Therapy

### Acyclovir

Complicated or severe varicella infections are typically treated with acyclovir as the first line of treatment. Acyclovir can be given either orally or intravenously depending on the case. Oral antiviral therapy with acyclovir is usually used for at-risk patients with the chickenpox and immunocompetent patients with herpes zoster. Due to the reduced bioavailability when given orally, immunocompromised patients with varicella infection should be treated with intravenous acyclovir. Depending on the route of administration, different side effects can be seen. If given orally, side effects involving the gastrointestinal system can be seen, whereas intravenous administration can cause neurological side effects. Any medications or drugs that have toxic effects on the kidneys are contraindicated when treating patients with acyclovir. Both liver and kidney functions should be watched carefully during treatment course.^2^

### Valaciclovir

An alternative to acyclovir is valaciclovir, a prodrug, which is taken orally and then activated via components of the patient’s metabolism. There is a significant increase in oral bioavailability when compared with that of acyclovir. Valaciclovir can be used in the treatment of adults with competent immune systems who are suffering from herpes zoster. However, it is not to be used for the treatment of children or adolescents. Gastrointestinal side effects can be seen after taking this antiviral therapy.^2^

### Famciclovir

The inactive form of penciclovir, famciclovir, is an antiviral therapy that is either used topically or taken orally. It acts as a nucleoside analog once activated by the body’s metabolism. Treatment of shingles in both immunocompromised and immunocompetent patients over the age of 25 years are the main uses for this particular antiviral. As with valaciclovir, this medication is not to be used to treat children or adolescents with varicella infections. Side effects can include nausea, headaches, and confusion.^2^

### Brivudin

This antiviral treatment has not been studied enough to have a full picture of its safety and uses. However, it can be used to treat adults with zoster infections who do not have any history of immunocompromise. Once again, this antiviral treatment has not been approved for use in individuals less than 25 years of age. Side effects with this antiviral therapy include reversible blood count changes, abnormal liver function tests, gastrointestinal distress, and impaired kidney function.^2^

### Foscarnet

Foscarnet does not require metabolism for activation and can be used against varicella strains that are resistant to treatment with nucleoside analogs. Its mechanism of action is the inhibition of DNA pol in viruses, which prevents exchange of pyrophosphate. Treatment with foscarnet is reserved for serious zoster infections in immunocompromised patients and VZV infections that appear to be acyclovir resistant. Two major side effects of foscarnet therapy are ulceration of mucosal membrane along the urogenital tract as well as impaired kidney function.^2^

### Other Therapies: Corticosteroids

Adhami and colleagues^33^ performed a retrospective cohort study of the use of corticosteroids in treatment of varicella pneumonia. Oxygenation was both rapidly and significantly improved when comparing patients with varicella pneumonia who were treated with corticosteroids with those who were not. Evidence showed no difference in length of hospital stay or amount of time spent mechanically ventilated. This study indicates that corticosteroid therapy with adjunct acyclovir therapy may be promising in patients with severe varicella pneumonia. However, future trials must be conducted to uncover the exact role the steroids play in treatment of varicella pneumonia.^33^

## Illustrative Case

**Figure 1.**
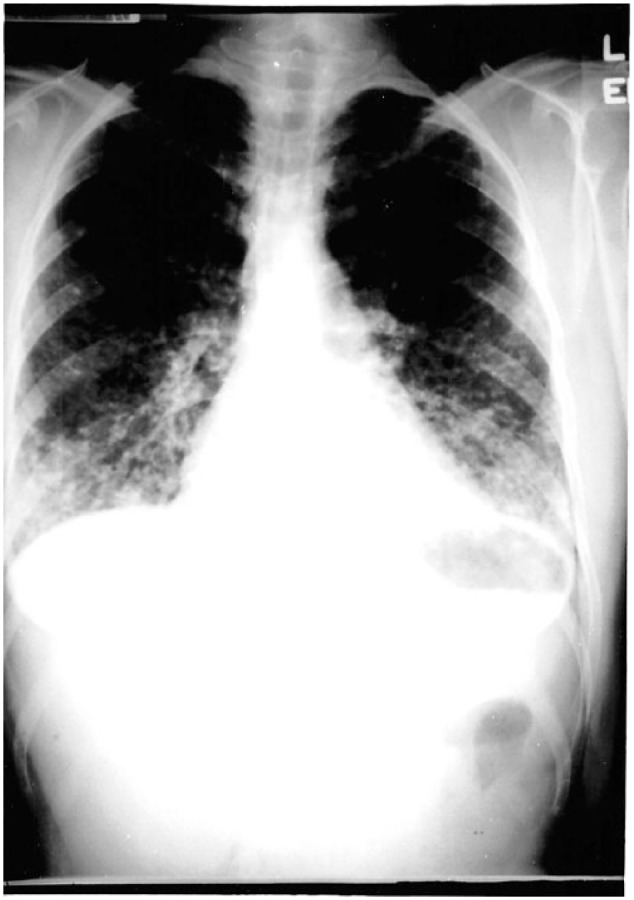
1st Chest radiograph (Outpatient).

A previously healthy 34-year-old female noted the development of a fever and rash consisting of vesicles over the past 4 days before being admitted to the hospital. Prior to onset the patient reports being generally well. While under outpatient care she became increasingly short of breath, was admitted, and then required transfer to the intensive care unit. Both her children acted as household contacts and exposed the patient to chickenpox.^34^ Figure 1 shows a chest radiograph taken in outpatient care.

Her past medical history included treatment for depression, for which she took buspirone and clomipramine hydrochloride. She was a cigarette smoker and reported smoking until her hospital admission.^34^

**Figure 2.**
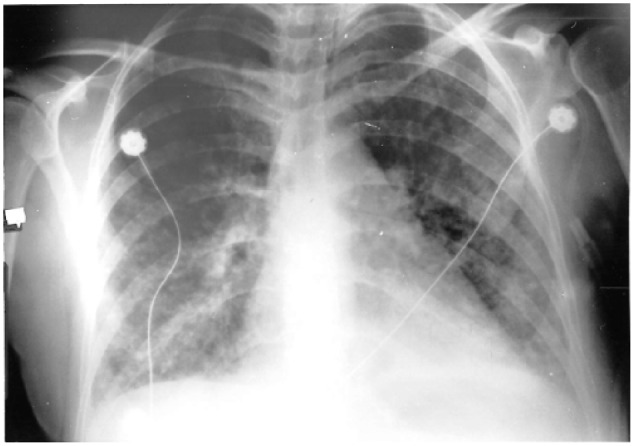
2nd Chest radiograph on hospital admission.

Vital signs showed a fever of 103.2°F, blood pressure of 130/60 mm Hg, elevated heart rate of 120 beats per minute, and significantly elevated respiratory rate of 30 breaths per minute. Physical examination of the skin was positive for vesicular rash in the distribution of the patient’s face, trunk, and extremities. Vesicles were filled with straw-colored fluid with a few encrusted vesicular lesions. Auscultation of the lungs revealed bilateral rales heard at the base and bilateral rhonchi. Cardiac examination yielded a regular S1, S2 rhythm with sinus tachycardia.^34^ Figure 2 shows a portable chest radiograph taken after the initial X-ray in Figure 1, which reveals a more prominent bilateral infiltrate of the interstitium with a nodular appearance.

Arterial blood gas was taken with the patient on 5 L of oxygen via nasal cannula. Results: pH 7.48, pCO_2_ 30, pO_2_ 88, HCO_3_ 22, and saturation at 96%. Antiviral treatment with acyclovir was initiated along with erythromycin to cover for any atypical microbes.

**Figure 3.**
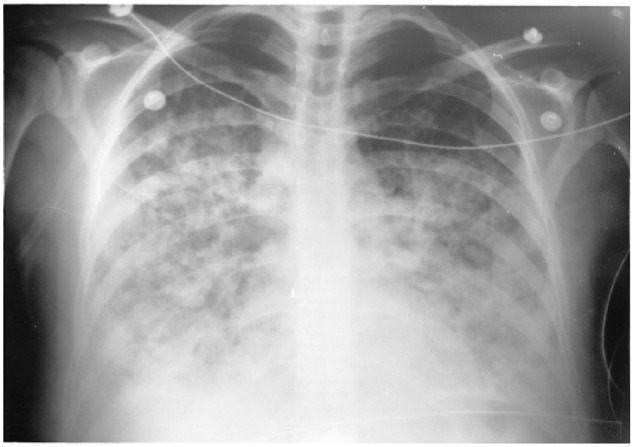
3rd Chest radiograph, done in ICU.

Despite therapy the patient experienced worsening tachypnea and reduced oxygen saturation with FIO_2_ of 100%. A third chest X-ray was performed and showed a ground-glass appearance consistent with adult respiratory distress syndrome (Figure 3).^34^

The patient’s refractory hypoxemia warranted discussion of intubation and mechanical ventilation. However, the patient’s pulse oximetry improved to 99% with a continuous positive airway pressure (CPAP) mask. CPAP was set at +10 cm H_2_O and humidified 100% oxygen and FIO_2_ was weaned to 50%. Sputum culture came back negative. After 4 days in the intensive care unit, the patient was stable on 6 L of oxygen via nasal cannula and transferred to the regular ward. The patient was discharged home on day 10 of admission following completion acyclovir treatment. On discharge, all vesicular lesions were encrusted, and pulse oximetry was 95% on room air. The patient was provided with a 21 mg transdermal nicotine patch before leaving the hospital. Final chest radiograph prior to discharge showed minimal improvement.^34^

## Discussion

The case report illustrates severe varicella pneumonia in a previously healthy, young adult without any history of immunosuppression. The only pertinent risk factor for this complication was the patient’s history of tobacco use. As stated previously, about 5% to 15% of adults who contract the chickenpox end up with some type of pulmonary complication.^23^ Advanced age, history of lung disease, immunosuppression, history of smoking, and pregnancy all place patients at risk of varicella progressing to pneumonia.^25^ However, severe complications have also occurred in immunocompetent, healthy individuals as well.^1^ The patient discussed only had one of these risk factors, smoking.^34^ Typically, as in this case, pulmonary symptoms occur less than a week following onset of rash and fever. Symptoms often include shortness of breath, persistent fever, and cough. Occasionally other symptoms like cyanosis, hemoptysis, and pleuritic chest pain can occur.^23^ Fortunately, this patient did not have many of the above-mentioned symptoms and responded to standard 10-day treatment with intravenous acyclovir. Other alternative drugs for treatment of varicella pneumonia were also reviewed. We were able to avoid an invasive intubation by utilizing CPAP therapy.^34^ Some evidence suggests that early diagnosis and initiation of treatment has a positive impact on patient prognosis with complications like pneumonia.^26^

The example case also illustrates the radiographic disease progression through the series of chest X-rays on the same patient.

## References

[1] Heininger U Desgrandchamps D Schaad UB. Seroprevalence of varicella-zoster virus IgG antibodies in Swiss children during the first 16 months of age. Vaccine. 2006;24:3258-3260. 16459000 10.1016/j.vaccine.2006.01.026

[2] Sauerbrei A. Diagnosis, antiviral therapy, and prophylaxis of varicella-zoster virus infections. Eur J Clin Microbiol Infect Dis. 2016;35:723-734. 26873382 10.1007/s10096-016-2605-0

[3] Heininger U Seward JF. Varicella. Lancet. 2006;368:1365-1376. 17046469 10.1016/S0140-6736(06)69561-5

[4] Gershon AA Steinberg SP Gelb L. Clinical reinfection with varicella-zoster virus. J Infect Dis. 1984;149:137-142. 6321605 10.1093/infdis/149.2.137

[5] Galil K Pletcher MJ Wallace BJ et al Tracking varicella deaths: accuracy and completeness of death certificates and hospital discharge records, New York State, 1989-1995. Am J Public Health. 2002;92:1248-1250. 12144978 10.2105/ajph.92.8.1248 PMC1447224

[6] Baxter R Tran TN Ray P et al Impact of vaccination on the epidemiology of varicella: 1995-2009. Pediatrics. 2014;134:24-30. 24913796 10.1542/peds.2013-4251

[7] Galil K Fair E Mountcastle N Britz P Seward J. Younger age at vaccination may increase risk of varicella vaccine failure. J Infect Dis. 2002;186:102-105. 12089668 10.1086/341089

[8] Arvin AM Gershon AA. Live attenuated varicella vaccine. Annu Rev Microbiol. 1996;50:59-100. 8905076 10.1146/annurev.micro.50.1.59

[9] Tyrrell DA. Clinical clues in virus infections. Br Med J. 1963;1:493-496. 13995046 10.1136/bmj.1.5329.493 PMC2123438

[10] Varicella and herpes zoster vaccines: WHO position paper, June 2014 [in French]. Wkly Epidemiol Rec. 2014;89:265-287. 24983077

[11] Thomas SL Hall AJ. What does epidemiology tell us about risk factors for herpes zoster? Lancet Infect Dis. 2004;4:26-33. 14720565 10.1016/s1473-3099(03)00857-0

[12] Habel LA Ray GT Silverberg MJ et al The epidemiology of herpes zoster in patients with newly diagnosed cancer. Cancer Epidemiol Biomarkers Prev. 2013;22:82-90. 23118142 10.1158/1055-9965.EPI-12-0815

[13] Yawn BP Wollan PC St Sauver JL Butterfield LC. Herpes zoster eye complications: rates and trends. Mayo Clin Proc. 2013;88:562-570. 23664666 10.1016/j.mayocp.2013.03.014 PMC3788821

[14] Ngai AL Staehle BO Kuter BJ et al Safety and immunogenicity of one vs two injections of Oka/Merck varicella vaccine in healthy children. Pediatr Infect Dis J. 1996;15:49-54. 8684876 10.1097/00006454-199601000-00011

[15] Stumpf MP Laidlaw Z Jansen VA. Herpes viruses hedge their bets. Proc Natl Acad Sci U S A. 2002;99:15234-15237. 12409612 10.1073/pnas.232546899 PMC137573

[16] Gustafson TL Lavely GB Brawner ER Jr Hutcheson RH Jr Wright PF Schaffner W. An outbreak of airborne nosocomial varicella. Pediatrics. 1982;70:550-556. 6289235

[17] Arvin AM. Varicella-zoster virus. Clin Microbiol Rev. 1996;9:361-381. 8809466 10.1128/cmr.9.3.361 PMC172899

[18] Ross AH. Modification of chicken pox in family contacts by administration of gamma globulin. N Engl J Med. 1962;267:369-376. 14494142 10.1056/NEJM196208232670801

[19] Nichols RA Averbeck KT Poulsen AG et al Household size is critical to varicella-zoster virus transmission in the tropics despite lower viral infectivity. Epidemics. 2011;3:12-18. 21420656 10.1016/j.epidem.2010.11.003 PMC3072572

[20] Abba AA Al-Khuwaitir TS Al-Moghairi AM Garg H. Presentation and outcome of varicella pneumonia in adults. Saudi Med J. 2005;26:338-340. 15770323

[21] Bonhoeffer J Baer G Muehleisen B et al Prospective surveillance of hospitalisations associated with varicella-zoster virus infections in children and adolescents. Eur J Pediatr. 2005;164:366-370. 15747132 10.1007/s00431-005-1637-8

[22] Askalan R Laughlin S Mayank S et al Chickenpox and stroke in childhood: a study of frequency and causation. Stroke. 2001;32:1257-1262. 11387484 10.1161/01.str.32.6.1257

[23] Voore N Lai R. Varicella pneumonia in an immunocompetent adult. CMAJ. 2012;184:1924. 22566523 10.1503/cmaj.111473 PMC3503906

[24] Almuneef M Memish ZA Balkhy HH Alotaibi B Helmy M. Chickenpox complications in Saudi Arabia: is it time for routine varicella vaccination? Int J Infect Dis. 2006;10:156-161. 16260166 10.1016/j.ijid.2005.02.008

[25] Tunbridge AJ Breuer J Jeffery KJ; British Infection Society. Chickenpox in adults—clinical management. J Infect. 2008;57:95-102. 18555533 10.1016/j.jinf.2008.03.004

[26] Alanezi M. Varicella pneumonia in adults: 13 years’ experience with review of literature. Ann Thorac Med. 2007;2:163-165. 19727368 10.4103/1817-1737.36551 PMC2732098

[27] Hunter J Stott SA. Life-threatening chickenpox pneumonitis in two previously healthy adults. J R Soc Med. 1999;92:472-474. 10645302 10.1177/014107689909200914 PMC1297364

[28] dos Santos MC Alecrim MG. Images in clinical medicine. Varicella pneumonia in an adult. N Engl J Med. 2010;362:1227. 20357285 10.1056/NEJMicm0902199

[29] Davis TA Angel J. Varicella pneumonia in pregnancy. Int J Obstet Anesth. 1997;6:274-278. 15321267 10.1016/s0959-289x(97)80036-2

[30] Sauerbrei A Wutzler P. Varicella during pregnancy. 2. Diagnosis, prevention and therapy [in German]. Dtsch Med Wochenschr. 2004;129:2045-2047. 15386208 10.1055/s-2004-831844

[31] Feldman S Hughes WT Daniel CB. Varicella in children with cancer: seventy-seven cases. Pediatrics. 1975;56:388-397. 1088828

[32] Jones AM Thomas N Wilkins EG. Outcome of varicella pneumonitis in immunocompetent adults requiring treatment in a high dependency unit. J Infect. 2001;43:135-139. 11676521 10.1053/jinf.2001.0874

[33] Adhami N Arabi Y Raees A Al-Shimemeri A Ur-Rahman M Memish ZA. Effect of corticosteroids on adult varicella pneumonia: cohort study and literature review. Respirology. 2006;11:437-441. 16771913 10.1111/j.1440-1843.2006.00870.x

[34] Ibrar S Denny JT. ARDS secondary to severe varicella pneumonia in an immunocompetent patient. Paper presented at: Society of Critical Care Anesthesiologists Annual Meeting; May 5, 2017; Washington, DC.

